# Supplementary material for: Thermochromic aggregation-induced dual phosphorescence via temperature-dependent sp3-linked donor-acceptor electronic coupling
Source: Nat Commun. 2021 Mar 1;12:1364. doi: 10.1038/s41467-021-21676-5 (PMC7921125; doi:10.1038/s41467-021-21676-5)
Supplement: Supplementary file 1 — Supplementary Information [file 41467_2021_21676_MOESM1_ESM.pdf]

Supplementary Information

Thermochromic Aggregation-Induced Dual Phosphorescence via Temperature-Dependent *sp*^{3}-Linked Donor-Acceptor Electronic Coupling

T. Wang *et al*.

Part I. Materials, methods, and synthesis.

Materials

Diphenylamine, 4-iodoanisole, 4-bromobenzonitrile, 4-cyanobenzyl bromide, 2, 2'-dipyridyl, 4-(bromomethyl)benzophenone and boron tribromide were purchased from Energy Chemical Co., Ltd. 4-(Diphenylamino)phenylboronic acid, 4'-bromoacetophenone, and 4-bromobenzophenone were obtained from Aladdin Reagent (Shanghai) Co., Ltd. Tetrakis(triphenylphosphine)palladium ($Pd(PPh_3)_4$) was acquired from Sigma-Aldrich LLC. All other reagents and solvents were acquired from Sinopharm Chemical Reagent Co., Ltd and used as received.

Theoretical Calculations.

Solution calculations. The ground-state (S_0) geometries for all the molecules studied in this work were optimized at the B3LYP[1]-D3(BJ)[2]/6-31G(d)[3] level. In THF, excited states were calculated using linear-response time-dependent density functional theory (TD-DFT) with optimally tuned LC-ωPBE*[4] range-separated functional and TZVP[5] basis set to simulate the absorption spectra, and the polarizable continuum model (PCM)[6] implicit solvation model was applied to take into account the effects of the solvent. The Gaussian broadening function with 0.5 eV Full Width at Half Maximum (FWHM) was employed to produce the theoretical absorption spectra. The hole-electron analysis for excited states was performed by Multiwfn 3.7(dev) program[7]. And the hole-electron and molecular orbital (MO) distributions were rendered by VMD 1.9.3 software.[8]

Vertical emission calculations. For **TPA1** in the gas state, the geometries of the S_1 and T_2 states were optimized using the implemented TD-DFT gradients at the CAM-B3LYP[9] /6-31G(d) level; the geometries of the T_1 states were assessed by spin-relaxed open-shell optimizations at the UCAM-B3LYP/6-31G(d) level. The reason we employed the CAM-B3LYP functional is related to the fact that B3LYP functional may overestimate electron delocalization in the excited states for donor-acceptor molecules. And the vertical emission energies of S_1, T_1, and T_2 were refined at the TD-LC-ωPBE*/TZVP level. The spin-orbit coupling (SOC) matrix elements between singlets and triplets were computed from TDDFT with spin-orbit mean-field (SOMF)[10] approach at the ωB97X-D3[11]/TZVP level using ORCA 4.2.1 program[12], in which the “grid4” DFT integration grid and “TightSCF” convergence criteria were employed.

Caculations of relative Gibbs free energies and the three global/local-minima geometries of T_1-state. For **TPA1** and **TPA4** molecules in the gas phase, the geometry optimization and frequency calculations of different T_1 and corresponding transition states were performed by spin-relaxed open-shell DFT method at the UM06-2X[13]-D3/6-31G(d) level. The frequency calculations confirmed that geometries of T_1 state were at a local minima and geometries of transition states were at a saddle point with an imaginary frequency. The electronic energies of T_1 and transition states were refined with a high-

level double-hybrid method at the PWPB95[14]-D3(BJ)/def2-QZVPP[15] level using ORCA 4.2.1 program[12], in which the RIJCOSX[16] approximation with def2/J auxiliary basis set[17] and "girdx4" COSX grid, and the "grid4" DFT integration with "TightSCF" convergence criteria were employed. The Gibbs free energy of T_1 and transition states were calculated by adding electronic energy and thermal correction to Gibbs free energy that obtained by frequency calculation. The vertical emission energies were calculated with TD-DFT method at the TD-M06-2X/TZVP level. The reason we employed the M06-2X functional for triplet excited state calculations is related to the fact that M06-2X functional has a better average accuracy in reproducing excitation energies than other functionals[18].

All the other DFT calculations were carried out by Gaussian 16 program[19]. The corresponding range-separation parameter (ω, in Bohr^{-1}) used in LC-ωPBE functional for each molecule was optimally-tuned according to the GAP-tuning method (see ref[20] for more details).

Synthesis

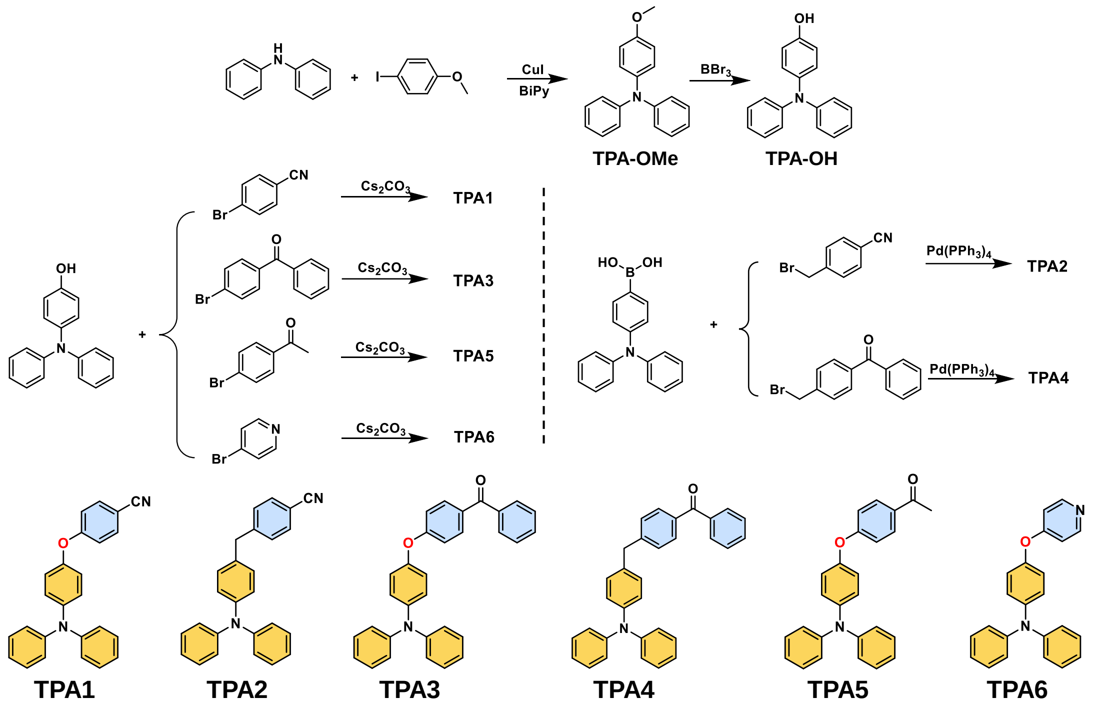

Supplementary Figure 1. Synthetic routes and chemical structures of AIE-active RTP molecules (**TPA1-6**).

Synthesis of 4-methoxy-*N*, *N*-diphenylaniline (**TPA-OMe**). Diphenylamine (1 equiv., 0.95 g), 4-iodoanisole (1.2 equiv., 1.34 g), 2,2'-dipyridyl (0.02 equiv., 0.018 g), CuI (0.02 equiv., 0.021 g) and potassium tert-butylate (t-BuOK, 1.5 equiv., 0.94 g) were added into a round-bottom flask containing 20 mL of toluene. The reaction was heated to reflux under N_2 for 4 h. After the reaction finished and cooled down to room temperature, the reaction solvent was wash by deionized water twice (30 mL×2). Then the organic layer was collected, and the solvent was removed by the rotary evaporator in vacuum. The

obtained crude product was further purified by column chromatography, giving the white solid (95%, 1.42 g).^{1}H NMR (400 MHz, DMSO-d_6), δ (TMS, ppm): 7.27- 7.21 (m, 4H), 7.04- 7.00 (m, 2H), 6.94 (dddt, J = 7.6, 6.4, 2.0, 1.0 Hz, 8H), 3.74 (s, 3H).

Synthesis of 4-(diphenylamino)phenol (**TPA-OH**). To a solution of **TPA-OMe** (1 equiv., 1.4 g) in dichloromethane (45 mL) was slowly added BBr_3 (1.5 equiv., 1.91 g) at 0 °C. The mixture was allowed to be stirred at 0 °C for 0.5 h under the protection of N_2, and then the system temperature was heated to 25 °C. After being stirred at 25 °C for 10 h, a saturated NH_4Cl aqueous solution (60 mL) was added into the reaction system to quench the reaction. Then the resulting mixture was extracted with dichloromethane three times (60 mL×3), and the organic layer was collected. The dichloromethane was removed by the rotary evaporator under reduced pressure, and the obtained crude product was purified by column chromatography, affording **TPA-OH** as the off-white solid (85%, 1.13 g). ^{1}H NMR (400 MHz, DMSO-d_6), δ (TMS, ppm): 7.27- 7.21 (m, 4H), 7.04- 7.00 (m, 2H), 6.94 (dddt, J = 7.6, 6.4, 2.0, 1.0 Hz, 8H), 3.74 (s, 3H).

Synthesis of 4-(4-(diphenylamino)phenoxy)benzonitrile (**TPA1**). **TPA-OH** (1 equiv., 0.32 g), 4-bromobenzonitrile (1.5 equiv., 0.33 g), caesium carbonate (Cs_2CO_3, 2 equiv., 0.80 g) were added into the round-bottom flask containing 4 mL of DMF. The reaction mixture was heated to reflux and allowed to react overnight. After the reaction finished, 20 mL of deionized water was added to quench the reaction. And then mixture was extracted with dichloromethane three times (20 mL×3). The organic layer was collected and removed under reduced pressure. The obtained crude product was purified by column chromatography, giving the white crystal (92%, 0.39 g). ^{1}H NMR (400 MHz, DMSO-d_6), δ (TMS, ppm): 9.40 (s, 1H), 7.26- 7.18 (m, 4H), 6.96- 6.87 (m, 8H), 6.79- 6.73 (m, 2H). HRMS (ESI) m/z: $[M]^+$ calcd for $C_{25}H_{18}ON_2$, 362.14136; found 362.14090.

Synthesis of 4-(4-(diphenylamino)benzyl)benzonitrile (**TPA2**). 150 mL of desgassed THF and 15 mL of Na_2CO_3 aqueous solution (2 mol/L) were added into a round-bottom flask and protected by N_2. Then 4-cyanobenzyl bromide (1 equiv., 0.8 g), 4-(diphenylamino)phenylboronic acid (1.2 equiv., 1.54 g), $Pd(PPh_3)_4$ (0.03 equiv., 0.15 g) were added to the flask. Then the reaction system was heated to reflux for 24 h. After the reaction system cooled down to room temperature, the solvent was removed under reduced pressure. Then the collected solid was re-dissolved in dichloromethane (100 mL) and washed with deionized water (100 mL) twice. The organic layer was collected and removed by the rotary evaporator in vacuum. The crude product was further purified by column chromatography to afford **TPA2** as the white solid (65%, 0.95 g). ^{1}H NMR (400 MHz, DMSO-d_6), δ (TMS, ppm): 7.80- 7.74 (m, 2H), 7.49-

7.44 (m, 2H), 7.30- 7.24 (m, 4H), 7.19- 7.15 (m, 2H), 7.03- 6.91 (m, 8H), 3.99 (s, 2H). HRMS (ESI) m/z: $[M]^+$ calcd for $C_{26}H_{20}N_2$, 360.16210; found 360.16137.

Synthesis of (4-(4-(diphenylamino)phenoxy)phenyl)(phenyl)methanone (**TPA3**). The synthetic method is similar to **TPA1** by replacing 4-bromobenzonitrile as 4-bromobenzophenone. The final product is the off-white crystal (93%, 0.50 g). ^{1}H NMR (400 MHz, DMSO-d_6), δ (TMS, ppm): 7.82- 7.77 (m, 2H), 7.74- 7.65 (m, 3H), 7.58- 7.53 (m, 2H), 7.34- 7.28 (m, 4H), 7.15- 7.01 (m, 12H). HRMS (ESI) m/z: $[M]^+$ calcd for $C_{31}H_{23}O_2N$, 441.17233; found 441.17193.

Synthesis of (4-(4-(diphenylamino)benzyl)phenyl)(phenyl)methanone (**TPA4**). The synthetic method is similar to **TPA2** by replacing 4-cyanobenzyl bromide as 4-(bromomethyl)benzophenone. The final product is the off-white crystal (63%, 0.98 g). ^{1}H NMR (400 MHz, DMSO-d_6), δ (TMS, ppm): 7.74- 7.65 (m, 5H), 7.55 (t, J = 7.6 Hz, 2H), 7.47- 7.42 (m, 2H), 7.30- 7.24 (m, 4H), 7.23- 7.18 (m, 2H), 7.04- 6.93 (m, 8H), 4.01 (s, 2H). HRMS (ESI) m/z: $[M]^+$ calcd for $C_{32}H_{25}ON$, 439.19307; found 439.19238.

Synthesis of 1-(4-(4-(diphenylamino)phenoxy)phenyl)ethan-1-one (**TPA5**). The synthetic method is similar to **TPA1** by replacing 4-cyanobenzyl bromide as 4-bromoacetophenone. The final product is the yellowish crystal (90%, 0.42 g). ^{1}H NMR (400 MHz, DMSO-d_6), δ (TMS, ppm): 8.01- 7.97 (m, 2H), 7.34- 7.28 (m, 4H), 7.10- 7.01 (m, 12H), 2.54 (s, 3H). HRMS (ESI) m/z: $[M]^+$ calcd for $C_{26}H_{21}O_2N$, 379.15668; found 379.15599.

Synthesis of *N*, *N*-diphenyl-4-(pyridin-4-yloxy)aniline (**TPA6**). The synthetic method is similar to **TPA1** by replacing 4-cyanobenzyl bromide as 4-bromopyridine. The final product is the white powder (87%, 0.36 g). ^{1}H NMR (400 MHz, DMSO-d_6), δ (TMS, ppm): 8.55- 8.42 (m, 2H), 7.37- 7.28 (m, 4H), 7.14- 7.02 (m, 10H), 6.99- 6.91 (m, 2H). HRMS (ESI) m/z: $[M+H]^+$ calcd for $C_{23}H_{19}ON_2$, 339.14919; found 339.14902.

Part II. Supplementary Figures and Tables.

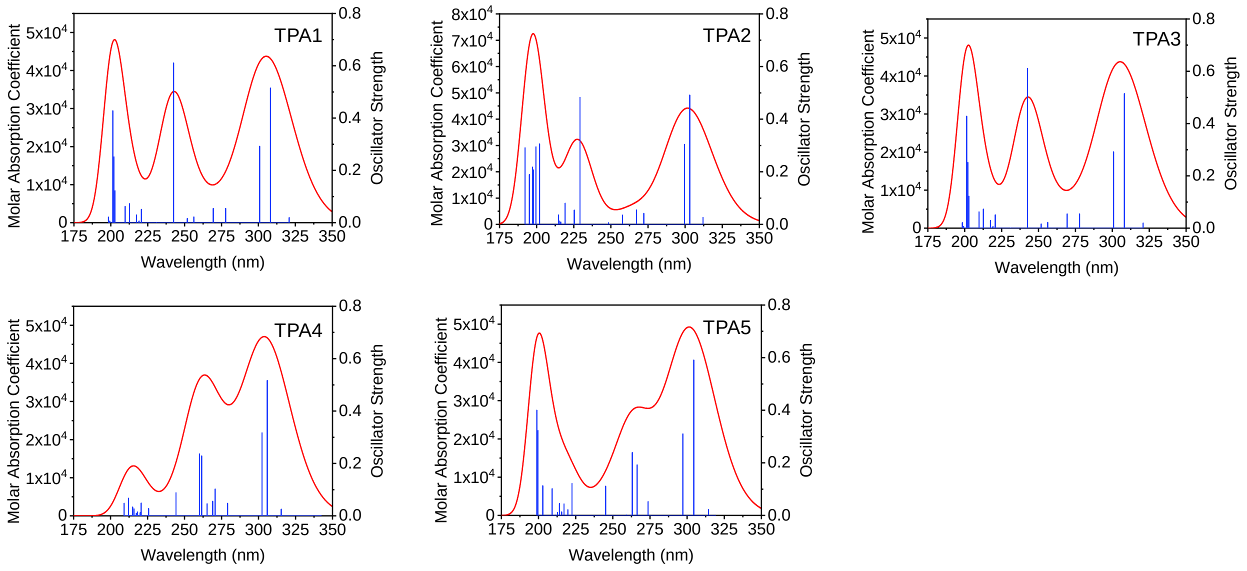

Supplementary Figure 2. Calculated absorption spectra of **TPA1-5** via TDDFT method with optimally tuning range-separated functional (LC-wPBE*) and TZVP basis set to simulate the absorption spectra.

Supplementary Table 1. Transition information of 4 lowest-lying excited states (maximum peak) in THF.

Moleclues	S_n	Energy/eV	Wavelength/nm	Oscillator Strength	Major (>5%) MO transitions
TPA1	1	3.87	320	0.0193	H -> L+1 62.2%, H -> L+3 21.5%, H -> L+4 5.1%, H -> L 5.0%
	2	4.03	308	0.5146	H -> L+2 93.8%
	3	4.12	300	0.2922	H -> L+4 75.5%, H -> L+3 19.7%
	4	4.47	277	0.0546	H -> L+5 67.2%, H -> L+3 13.4%
TPA2	1	3.98	311	0.0263	H -> L+1 54.1%, H -> L+2 28.0%, H -> L+4 9.4%
	2	4.09	302	0.4915	H -> L+2 45.8%, H -> L+4 35.9%, H -> L 7.1%
	3	4.14	299	0.3044	H -> L+3 92.4%
	4	4.56	271	0.0422	H -> L+5 48.2%, H -> L+6 14.2%, H -> L+4 9.6%, H -> L 9.1%
TPA3	1	3.66	339	0.0024	H-2 -> L 77.2%, H-1 -> L 8.2%, H-8 -> L 6.7%
	2	3.89	318	0.0229	H -> L+1 75.4%, H -> L+4 8.4%, H -> L+2 6.7%
	3	4.01	309	0.7399	H -> L+2 63.6%, H -> L+4 9.6%, H -> L 7.7%, H-1 -> L 6.2%
	4	4.13	300	0.3016	H -> L+3 93.9%
TPA4	1	3.63	341	0.0018	H-1 -> L 46.6%, H-2 -> L 34.3%, H-9 -> L 9.3%
	2	3.93	315	0.0251	H -> L+1 86.7%
	3	4.05	305	0.5170	H -> L+3 46.0%, H -> L+2 31.8%, H -> L+5 8.0%, H -> L 5.6%
	4	4.10	302	0.3171	H -> L+4 89.7%
TPA5	1	3.88	319	0.0002	H-2 -> L 92.6%
	2	3.95	314	0.0219	H -> L+1 82.0%, H -> L+4 7.9%
	3	4.07	304	0.5909	H -> L+2 83.6%, H -> L 6.5%
	4	4.18	296	0.3102	H -> L+3 93.7%

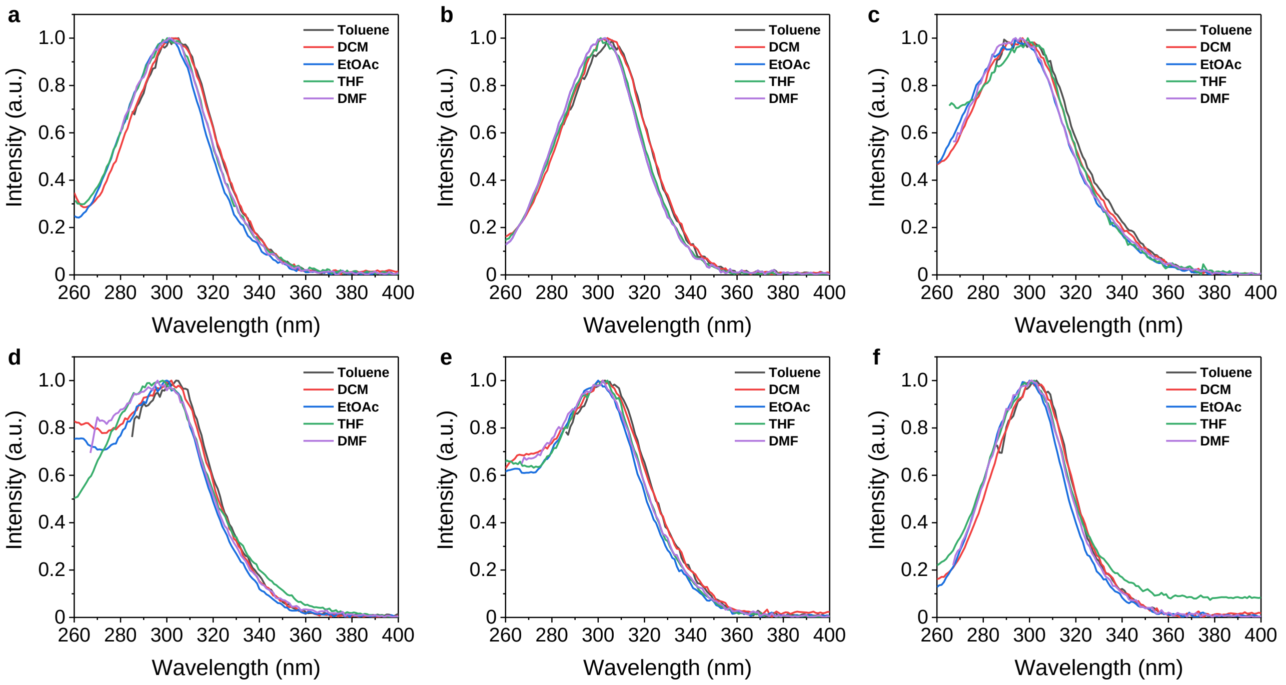

Supplementary Figure 3. UV-vis absorption spectra of (a) TPA1, (b) TPA2, (c) TPA3, (d) TPA4, (e) TPA5, and (f) TPA6 in various optically dilute solvents.

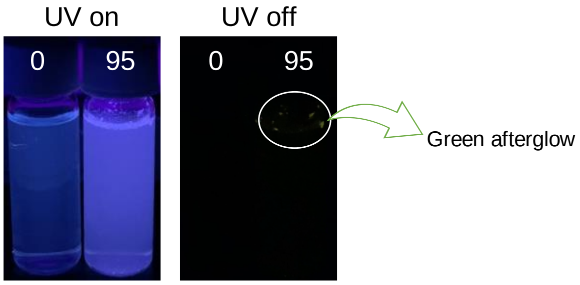

Supplementary Figure 4. AIE photos of **TPA1** with water/THF ratio of 95/5 (v/v) under 356-nm excitation. (concentration: 2.0 ×10^{-3} mol/L).

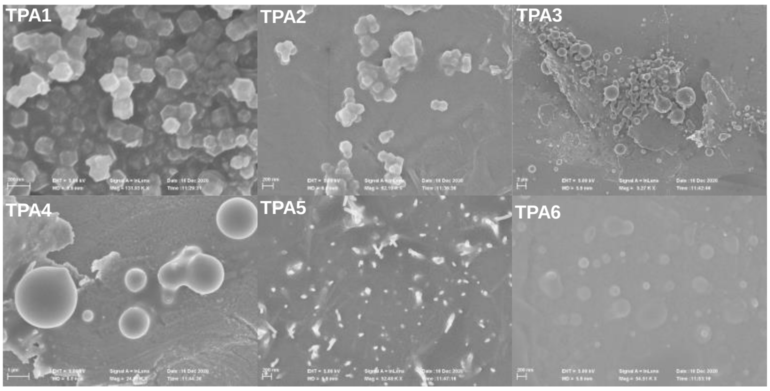

Supplementary Figure 5. Scanning electron microscopy images of **TPA1-6**.

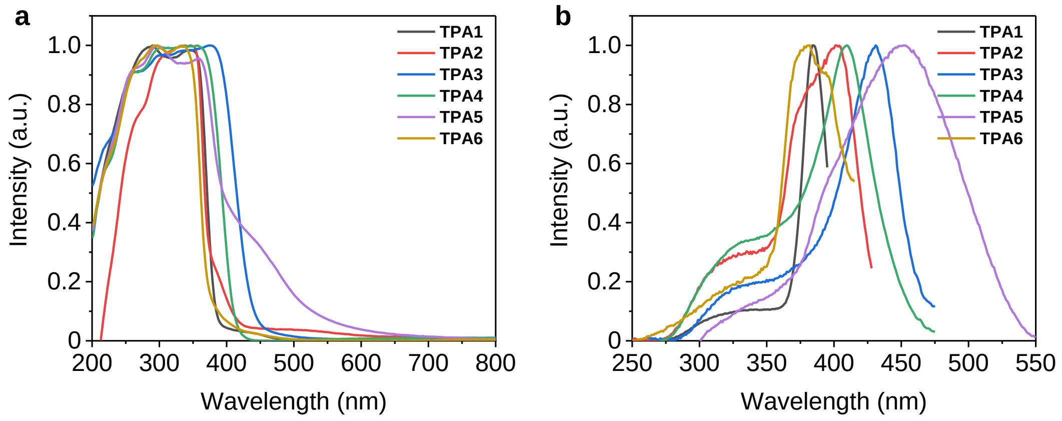

Supplementary Figure 6. (a) Solid-state UV-vis absorption and (b) excitation spectra of **TPA1-6**.

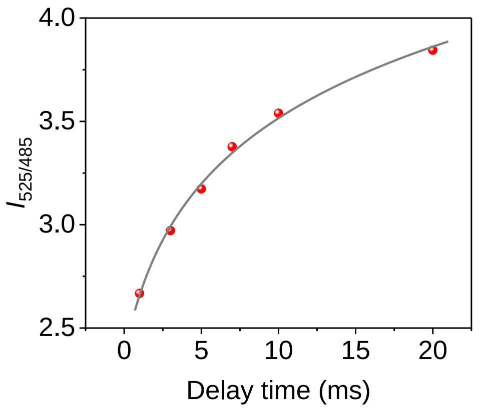

Supplementary Figure 7. Variation of the emission intensity ratio of **TPA1** between 485 and 525 nm at a different delay time at room temperature.

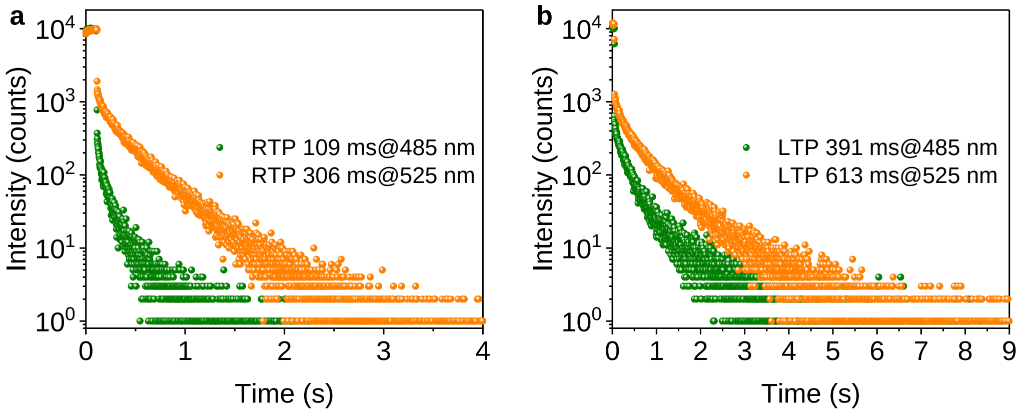

Supplementary Figure 8. RTP (a) and LTP (b, at 77 K) decay profiles of **TPA1**.

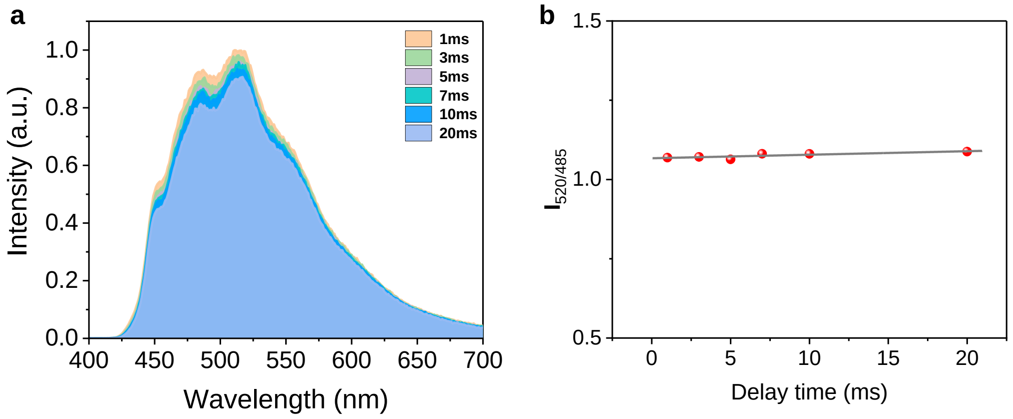

Supplementary Figure 9. (a) Time-resolved emission spectra of **TPA1** in air at 77 K (excitation:365 nm). (b) Variation of the emission intensity ratio of **TPA1** between 485 and 525 nm at a different delay time at 77 K.

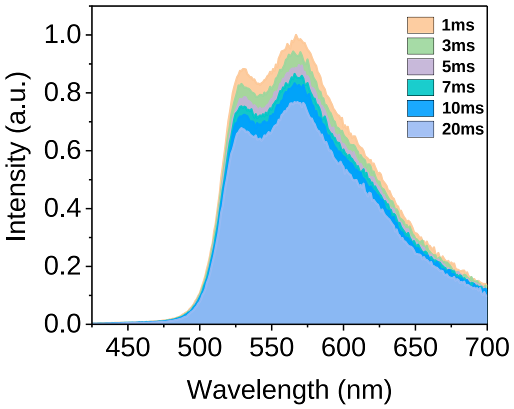

Supplementary Figure 10. Time-resolved emission spectra of **TPA2** in air at room temperature (excitation:365 nm).

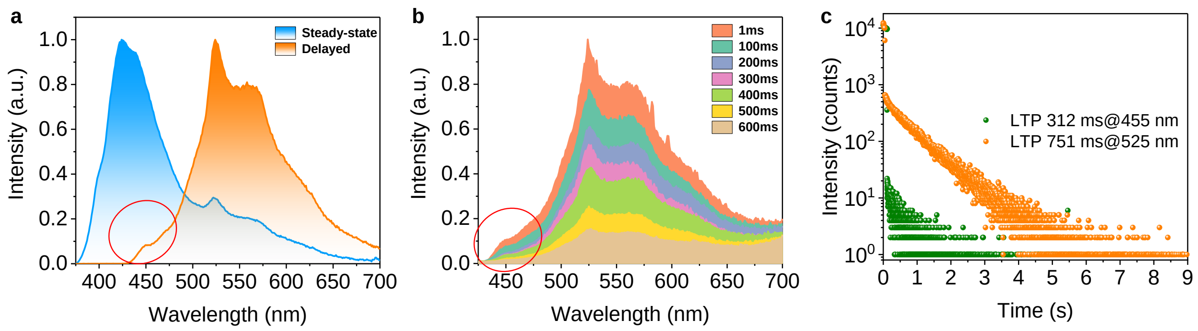

Supplementary Figure 11. (a) Steady-state and delayed emission spectra of **TPA2** in vacuum at 77 K (Δt= 3 ms). (b) Time-resolved emission spectra of **TPA2** in vacuum at 77 K (excitation: 365 nm). (c) LTP decay profiles of **TPA2** at 77 K.

Supplementary explanation for Supplementary Figure 11:

In contrast to steady-state emission of **TPA2** at room temperature, low-temperature (77 K) emission spectrum shows more structured profiles due to inhibition of the specific vibrational energy levels. It is worth noting that a weak emission band around 425-485 nm shows up in low-temperature phosphorescence (LTP) emission, the lifetime of which is up to 312 ms, shorter than that of T_1 emission (Supplementary Figure 11c, 751 ms). Given subsecond-scale luminescence and large energy gap (8.9 kcal/mol) between this long-lived emissive state (455 nm) and T_1 (~525 nm), it seems impossible that long-lived emission pertains to delayed fluorescence at 77 K. Here low-temperature photoluminescence could be attributed to prompt fluorescence and two-state phosphorescence.

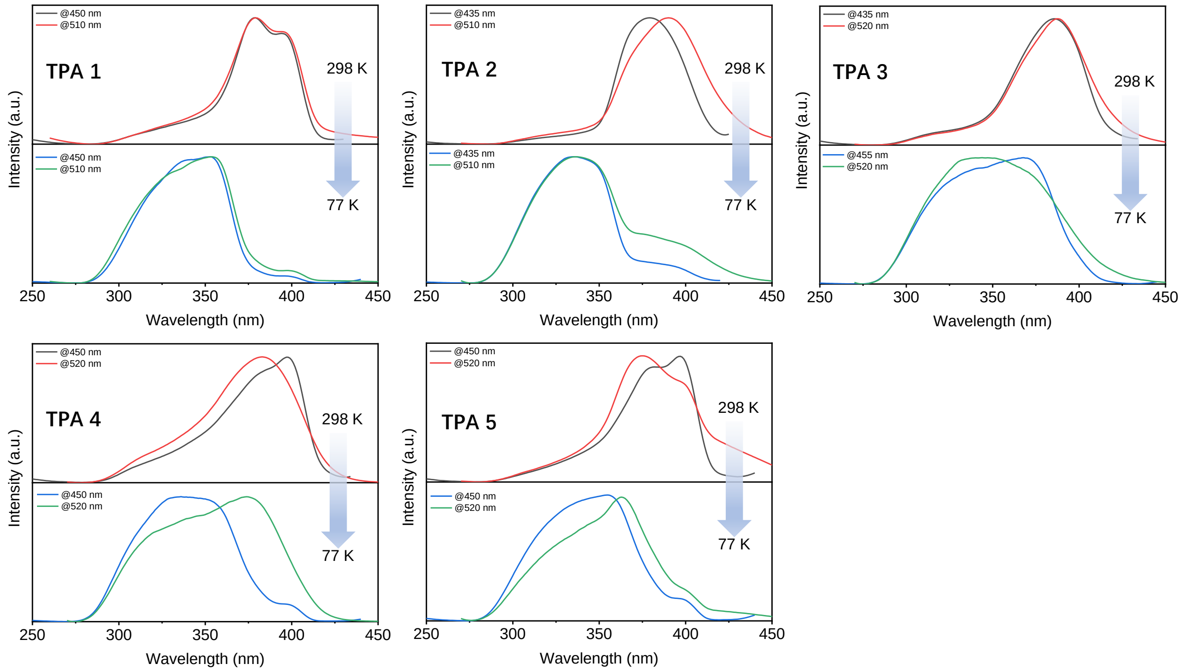

Supplementary Figure 12. Excitation spectra of **TPA1-5** dissolved in PMMA films at room temperature and 77 K showing inhibition of internal conversion at low temperature.

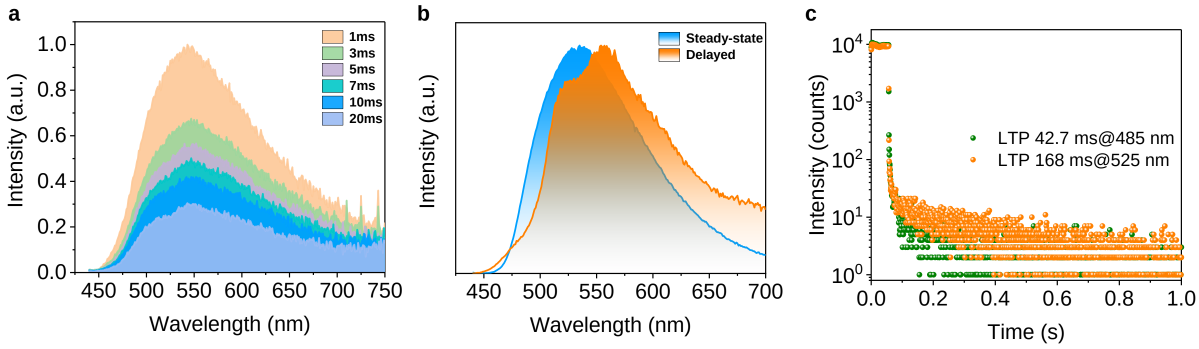

Supplementary Figure 13. (a) Time-resolved emission spectra of **TPA3** in air at room temperature. (b) Steady-state and delayed emission spectra 77 K (excitation:430 nm). (c) Time-resolved decay profiles of

TPA3 at 77 K.

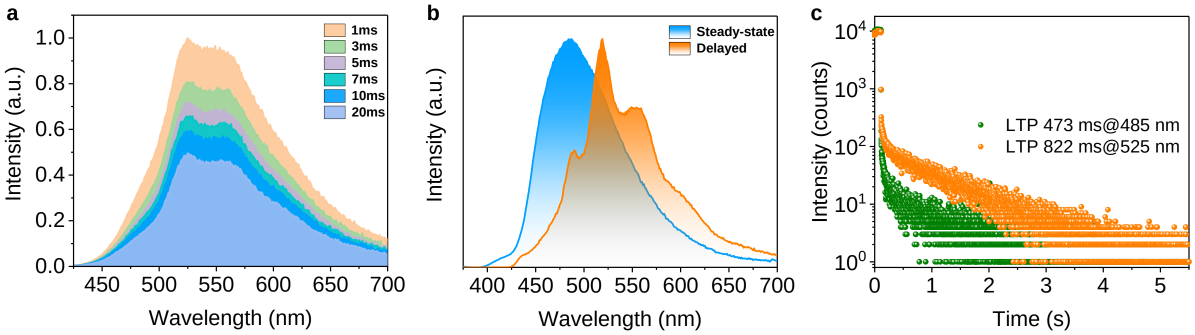

Supplementary Figure 14. (a) Time-resolved emission spectra of **TPA4** in air at room temperature. (b) Steady-state and delayed emission spectra 77 K (excitation:365 nm). (c) Time-resolved decay profiles of **TPA4** at 77 K.

Supplementary explanation for Supplementary Figure 14:

Time-resolved emission spectra at room temperature and delayed emission at 77 K both show an emission band around 450-500 nm. Similar to **TPA1**, this emission also belongs to the higher triplet energy level. It has to be noted that the emission band (~400-425nm) mainly results from S_1 because of a fluorescence lifetime can be collected (Supplementary Table 2).

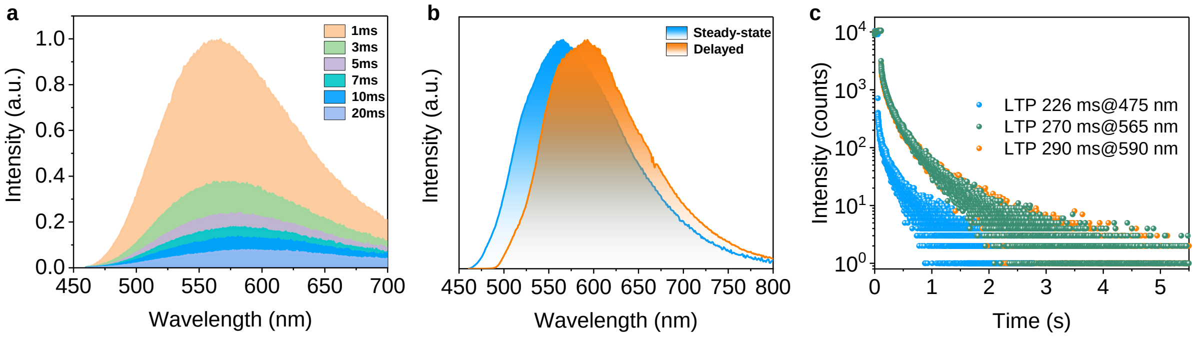

Supplementary Figure 15. (a) Time-resolved emission spectra of **TPA5** in air at room temperature. (b) Steady-state and delayed emission spectra 77 K (excitation:450 nm). (c) Time-resolved decay profiles of **TPA5** at 77 K.

Supplementary Table 2 Summarized emission lifetime and quantum yield data of **TPA1-5**.

**Compound**	**Fluorescence[a] (ns)**	**RTP[b] (ms)**	**LTP[c] (ms)**	**F/%**	
				FL[d]	RTP[e]

**TPA1**	1.0@403 nm	109@485 nm<br>306@525 nm	391@485 nm<br>613@525 nm	21.7	9.5
**TPA2**	1.8@437 nm	340@568 nm	312@455 nm<br>751@525 nm	39.8	10
**TPA3**	5.0@450 nm	1.7@527 nm<br>7.6@545 nm	42.7@485 nm<br>168@525 nm	0.3	8.2
**TPA4**	1.1@420 nm	68.4@510 nm<br>77.8@525 nm	473@485 nm<br>822@585 nm	3.5	13.3
**TPA5**	0.6@480 nm	31.1@565 nm<br>36.3@590 nm	226@475 nm<br>270@565 nm<br>290@ 590 nm	<0.2	13.6

a. Fluorescence lifetime at 298 K in air. b. RTP lifetime at 298 K in air. c. Phosphorescence lifetime at 77 K. d. Fluorescence quantum yield at 298 K in air. e. RTP quantum yield at 298 K in air.

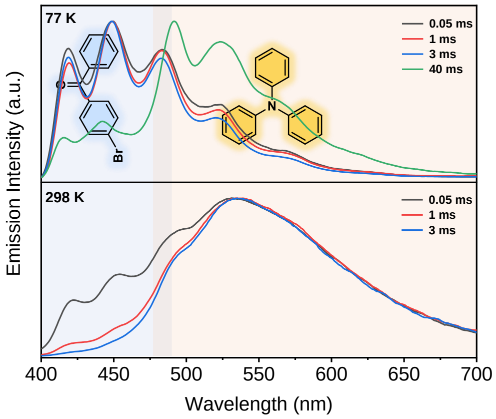

Supplementary Figure 16. Phosphorescence emission spectra of the binary mixture between **TPA** and 4-bromo-benzophenone (molar ratio = 1:1) at 298 K and 77 K with different delay time.

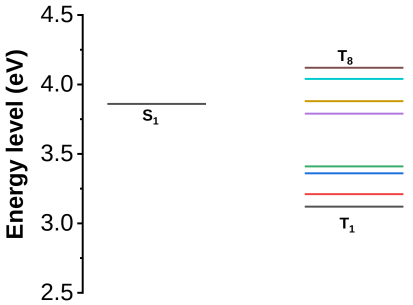

	S_1 (3.86 eV)
T_1 (3.12 eV)	1.21
T_2 (3.21 eV)	1.06
T_3 (3.36 eV)	0.27
T_4 (3.41 eV)	0.66
T_5 (3.79 eV)	0.16
T_6 (3.88 eV)	0.71
T_7 (4.04 eV)	0.63
T_8 (4.12 eV)	0.72

Supplementary Figure 17. Calculated energy diagram of vertical excitations of **TPA1** based on TD-LC-wPBE*/TZVP with S_0 optimized at B3LYP-D3(BJ)/6-31G(d) level. Tables show the spin-orbit coupling constants of S_1 and T_n.

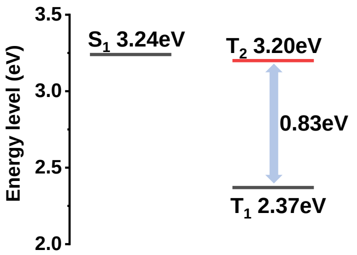

Supplementary Figure 18. Theoretical calculations of vertical emission energy levels of **TPA1** based on optimized S_1, T_1 and T_2, respectively.

Supplementary Table 3 Structure data of single crystals of **TPA1**, **TPA3**, **TPA4**, **TPA5**.

Compound	**TPA1**	**TPA3**	**TPA4**	**TPA5**
Empirical formula	$C_{25}H_{18}N_2O$	$C_{31}H_{23}NO_2$	$C_{32}H_{25}NO$	$C_{26}H_{21}NO_2$
Formula weight	362.41	441.50	439.53	379.44
Temperature/K	293(2)	293(2)	293(2)	293(2)
Crystal system	orthorhombic	monoclinic	monoclinic	monoclinic
Space group	Pbca	$P2_1/c$	$P2_1/c$	$P2_1$
a/Å	18.23812(11)	11.61402(10)	9.79540(10)	7.61340(10)
b/Å	9.24939(6)	22.77433(17)	24.8878(3)	23.1452(2)
c/Å	22.78821(14)	9.03780(8)	9.88320(12)	11.49810(10)
α/°	90	90	90	90
β/°	90	101.6329(8)	94.2720(10)	93.8140(10)
γ/°	90	90	90	90
Volume/$Å^3$	3844.18(4)	2341.41(3)	2402.69(5)	2021.64(4)
Z	8	4	4	4
Density(g/cm^3)	1.252	1.252	1.215	1.247
μ/mm^{-1}	0.605	0.612	0.561	0.620
F(000)	1520.0	928.0	928.0	800.0

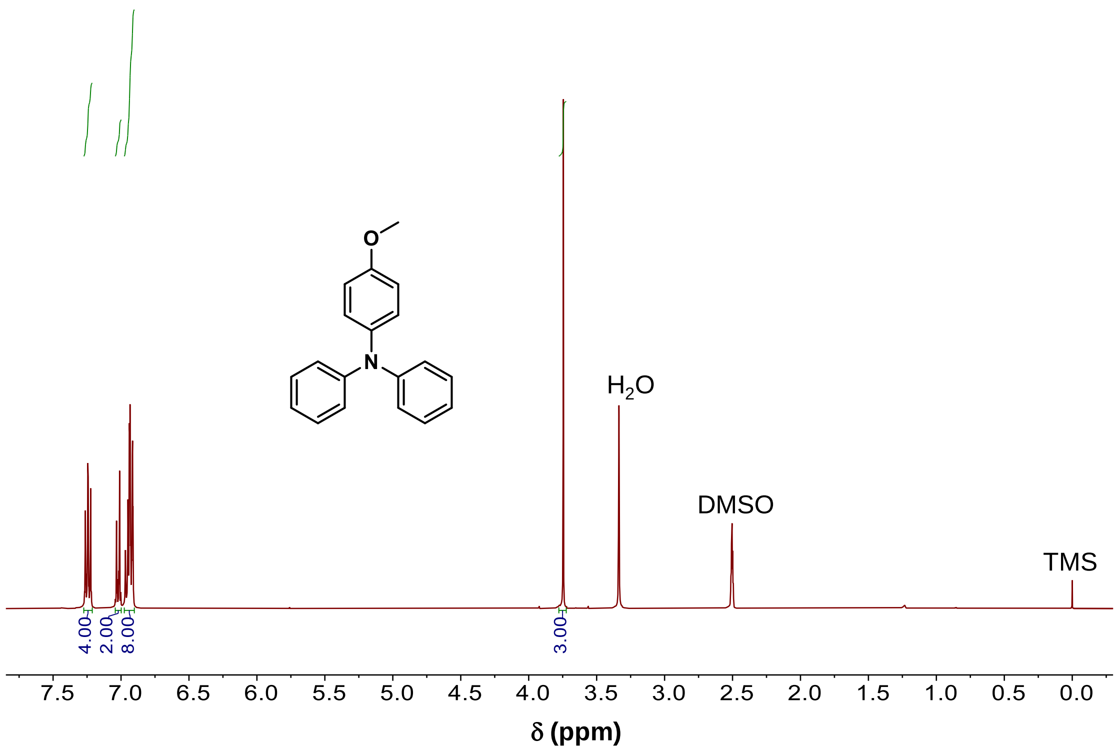

Supplementary Figure 18. ^{1}H NMR spectrum of **TPA-OMe**.

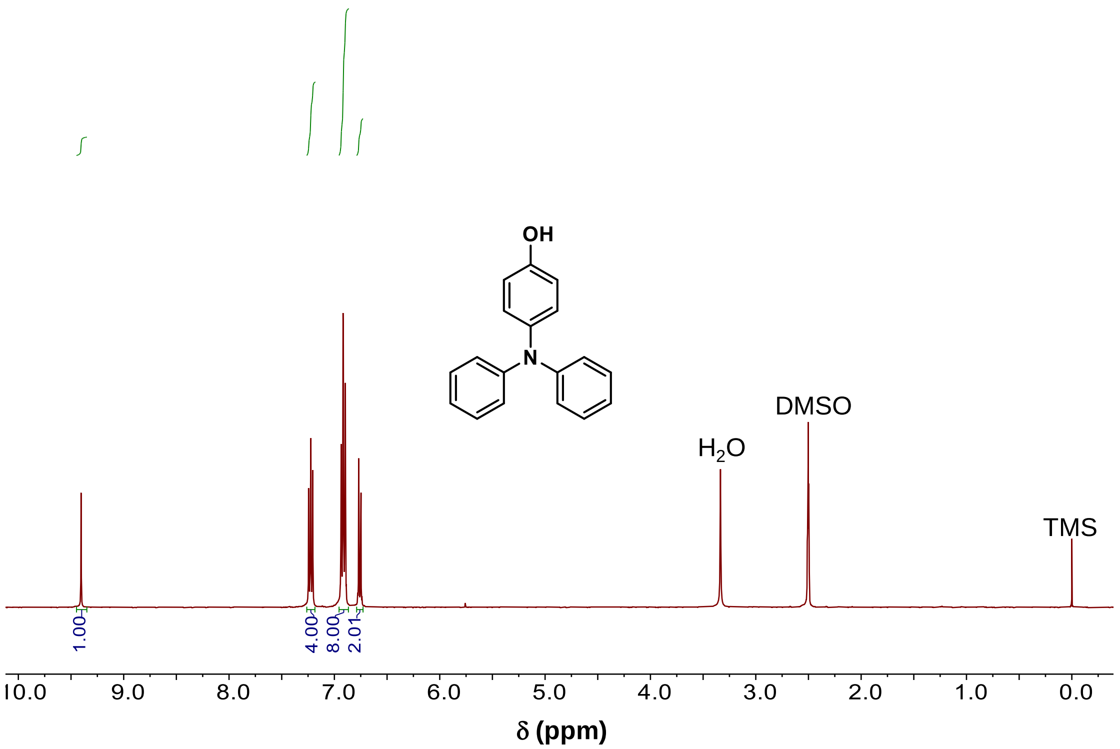

Supplementary Figure 19. ^{1}H NMR spectrum of **TPA-OH**.

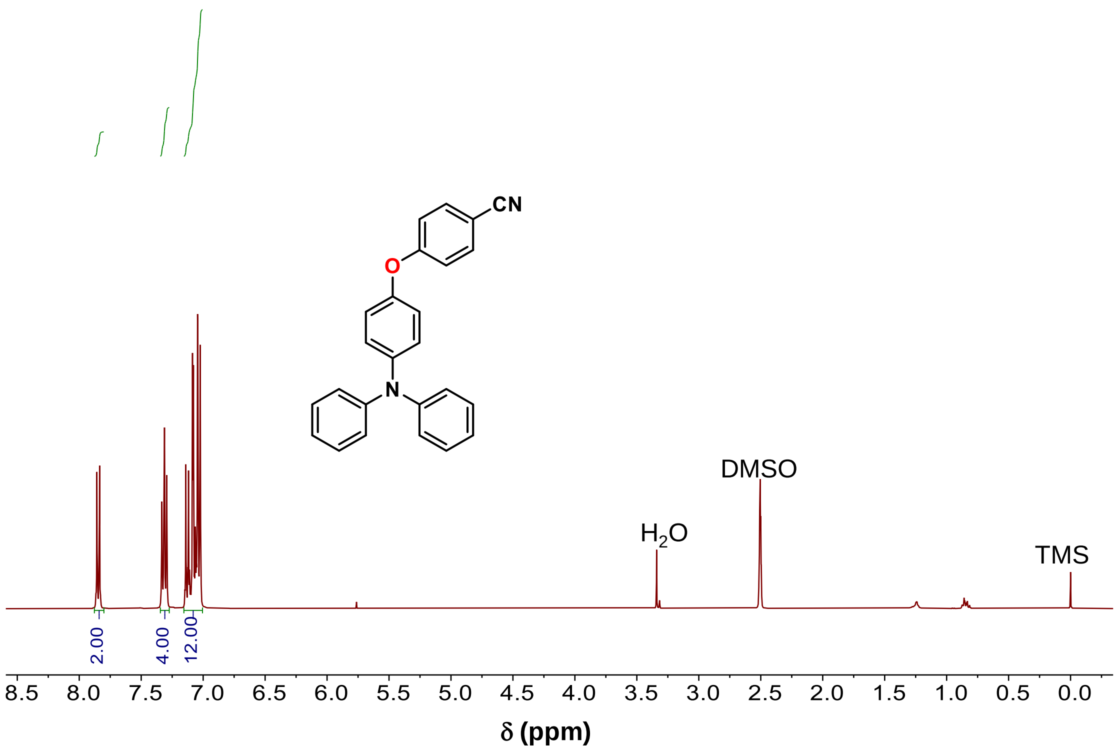

Supplementary Figure 20. ^{1}H NMR spectrum of **TPA1**.

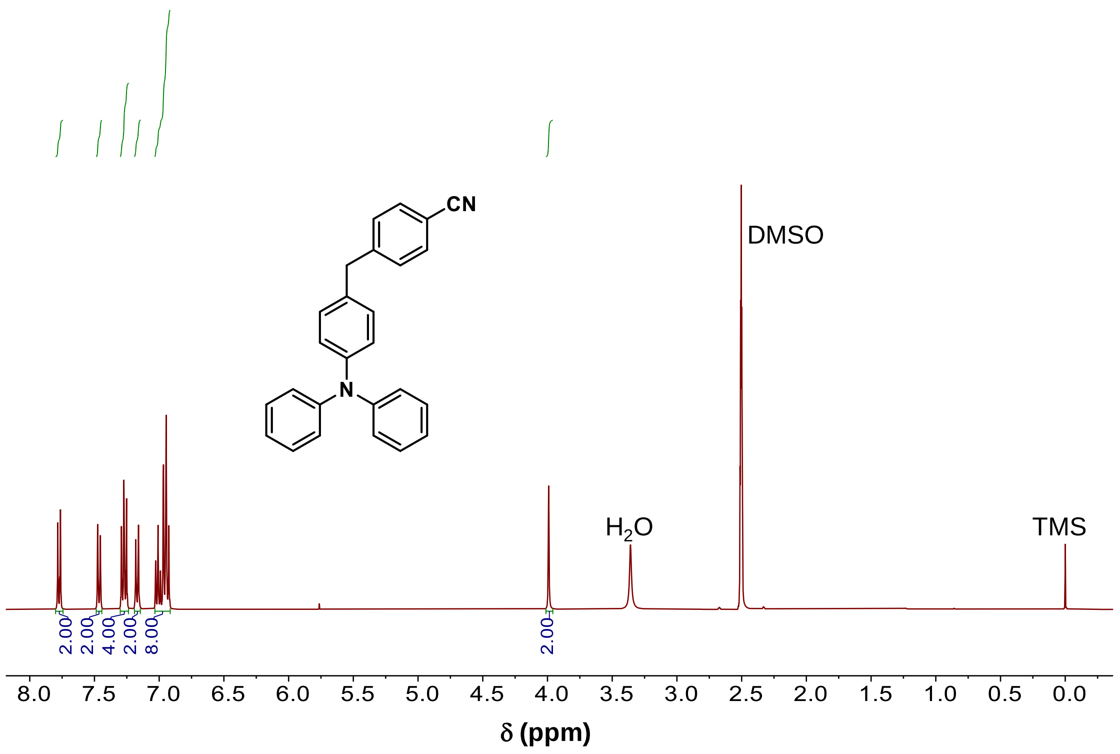

Supplementary Figure 21. ^{1}H NMR spectrum of **TPA2**.

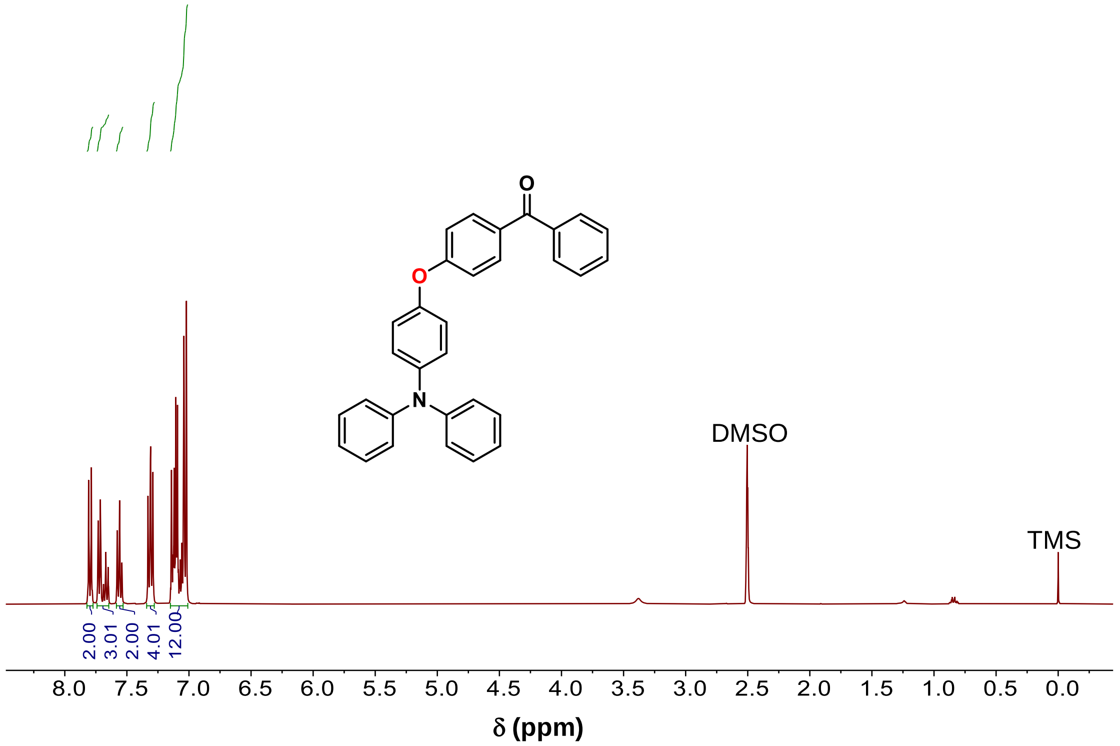

Supplementary Figure 22. ^{1}H NMR spectrum of **TPA3**.

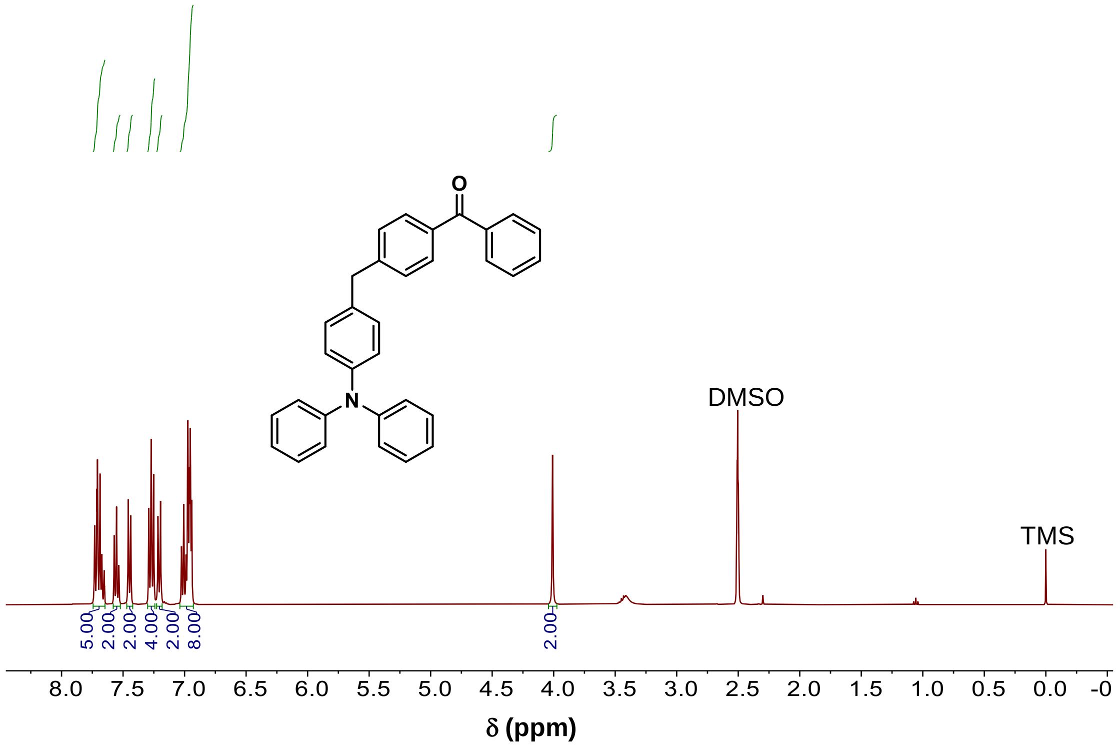

Supplementary Figure 23. ^{1}H NMR spectrum of **TPA4**.

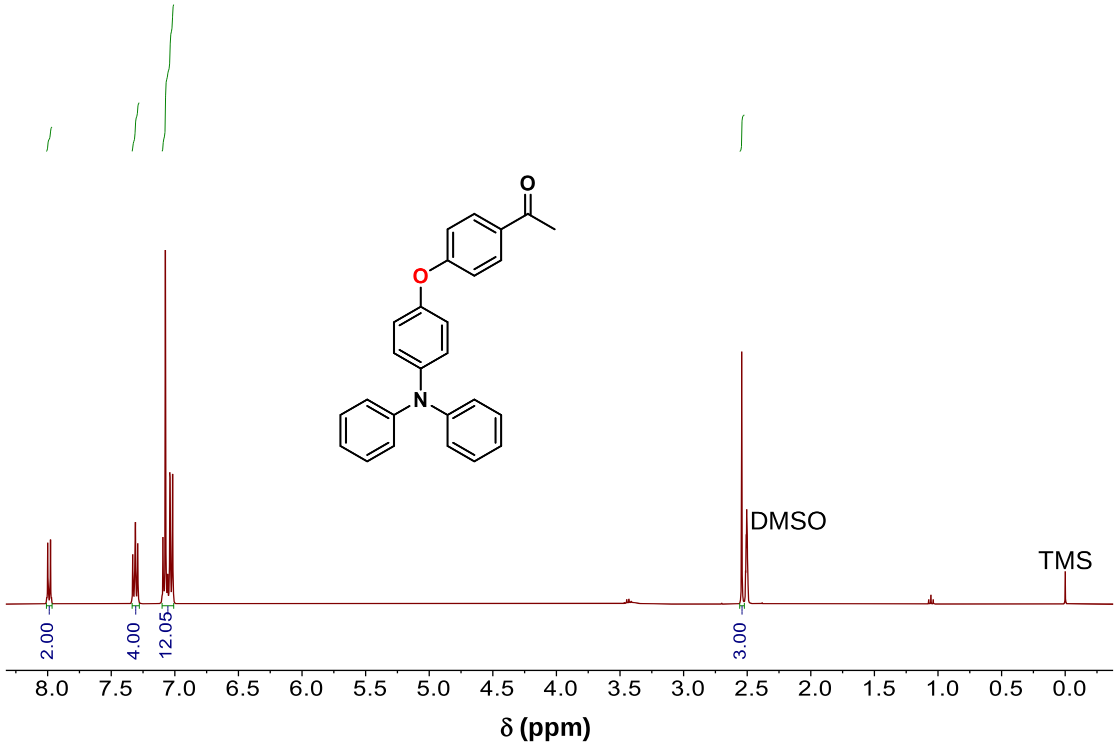

Supplementary Figure 24. ^{1}H NMR spectrum of **TPA5**.

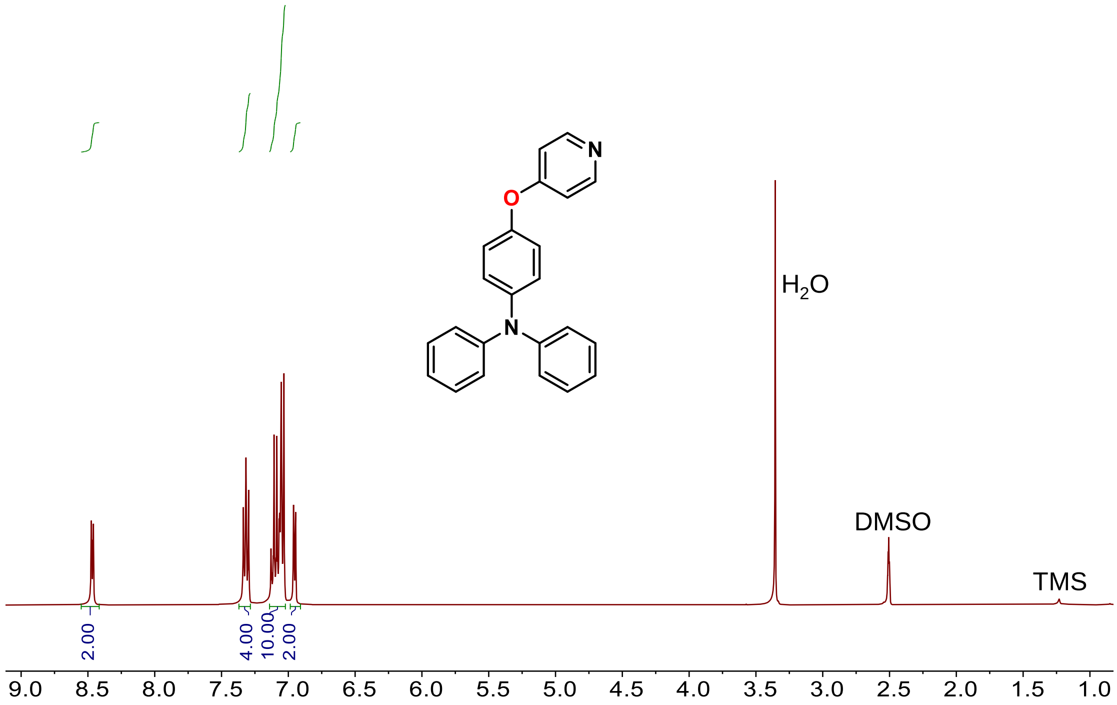

Supplementary Figure 25. ^{1}H NMR spectrum of **TPA6**.

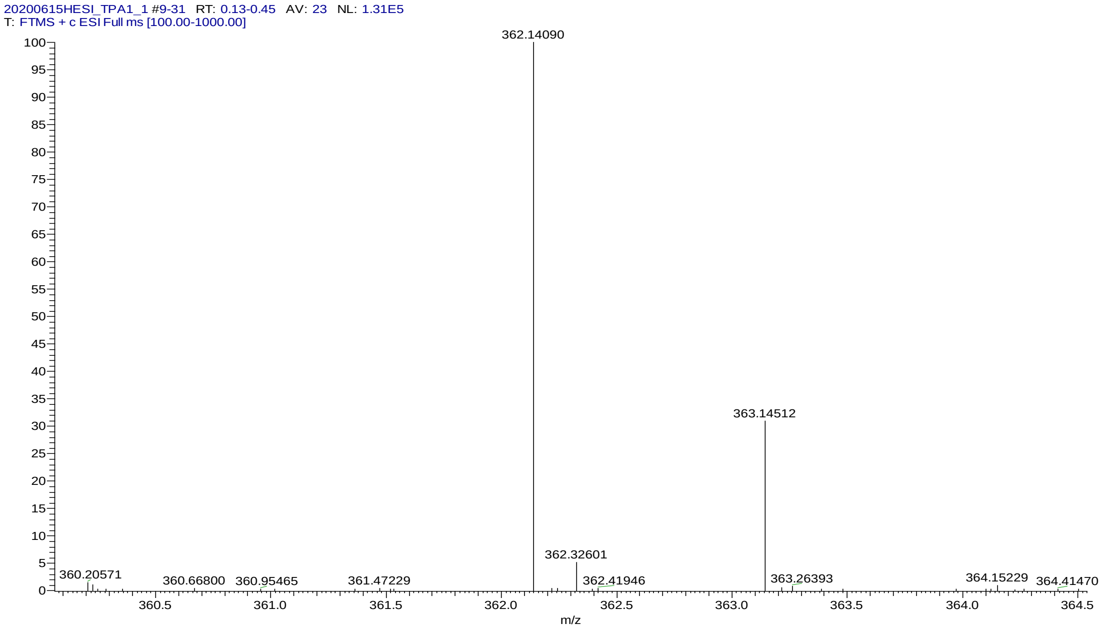

m/z	Intensity	Relative	Theo. Mass	Delta (mmu)	RDB equiv.	Composition
362.1409	130839.1	100	362.14136	-0.47	18	C25 H18 O N2
363.14512	40547.3	30.99	363.14472	0.4	18	C24 [13]C H18 O N2

Supplementary Figure 26. ESI mass spectrum of **TPA1**.

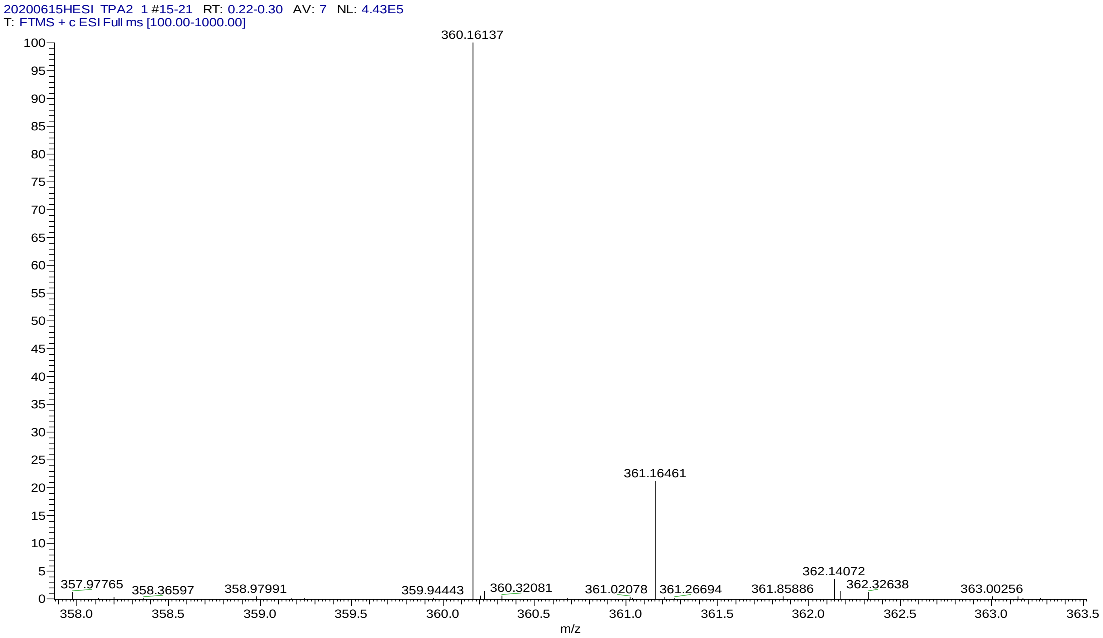

m/z	Intensity	Relative	Theo. Mass	Delta (mmu)	RDB equiv.	Composition
360.161	442816.1	100	360.1621	-0.73	18	C26 H20 N2
361.165	93871.4	21.2	361.1655	-0.85	18	C25 [13]C H20 N2

Supplementary Figure 27. ESI mass spectrum of **TPA2**.

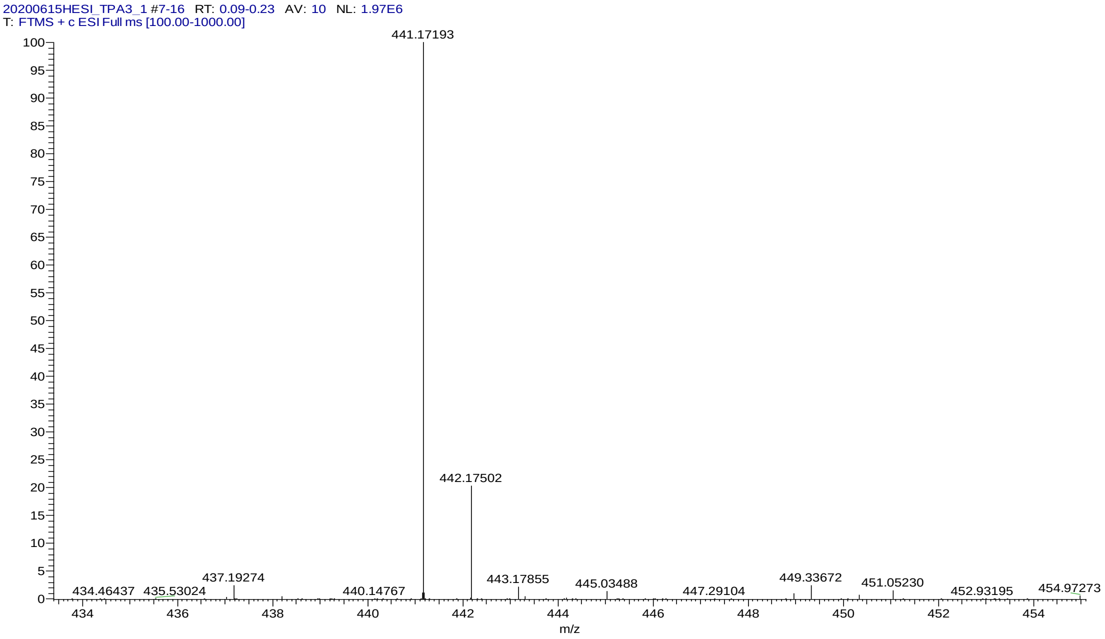

m/z	Intensity	Relative	Theo. Mass	Delta (mmu)	RDB equiv.	Composition
441.172	1974432	100	441.1723	-0.4	21	C31 H23 O2 N
442.175	400597	20.29	442.1757	-0.67	21	C30 [13]C H23 O2 N

Supplementary Figure 28. ESI mass spectrum of **TPA3**.

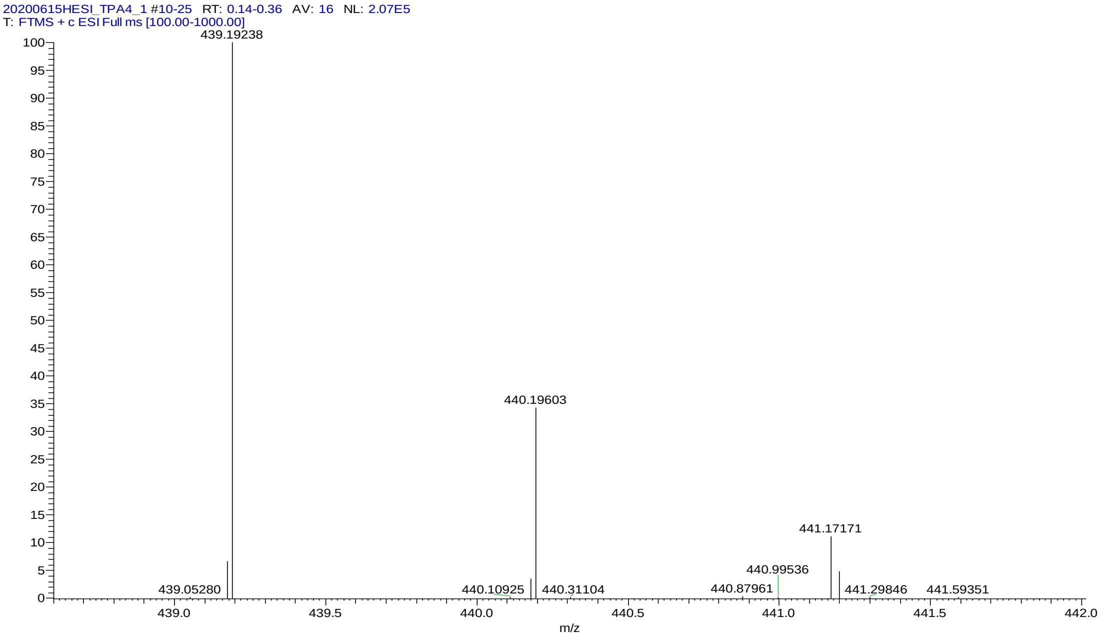

m/z	Intensity	Relative	Theo. Mass	Delta (mmu)	RDB equiv.	Composition
439.19238	206657.9	100	439.19307	-0.69	21	C32 H25 O N
440.19603	70756.3	34.24	440.19642	-0.4	21	C31 [13]C H25 O N

Supplementary Figure 29. ESI mass spectrum of **TPA4**.

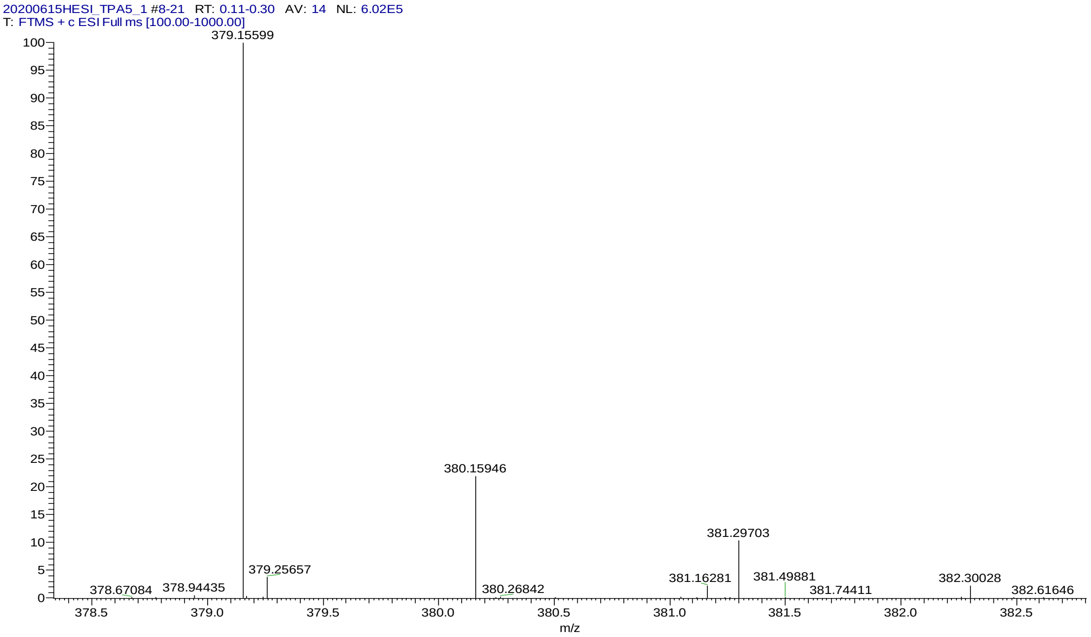

m/z	Intensity	Relative	Theo. Mass	Delta (mmu)	RDB equiv.	Composition
379.156	601862	100	379.1567	-0.69	17	C26 H21 O2 N
380.159	131937	21.92	380.16	-0.57	17	C25 [13]C H21 O2 N

Supplementary Figure 30. ESI mass spectrum of **TPA5**.

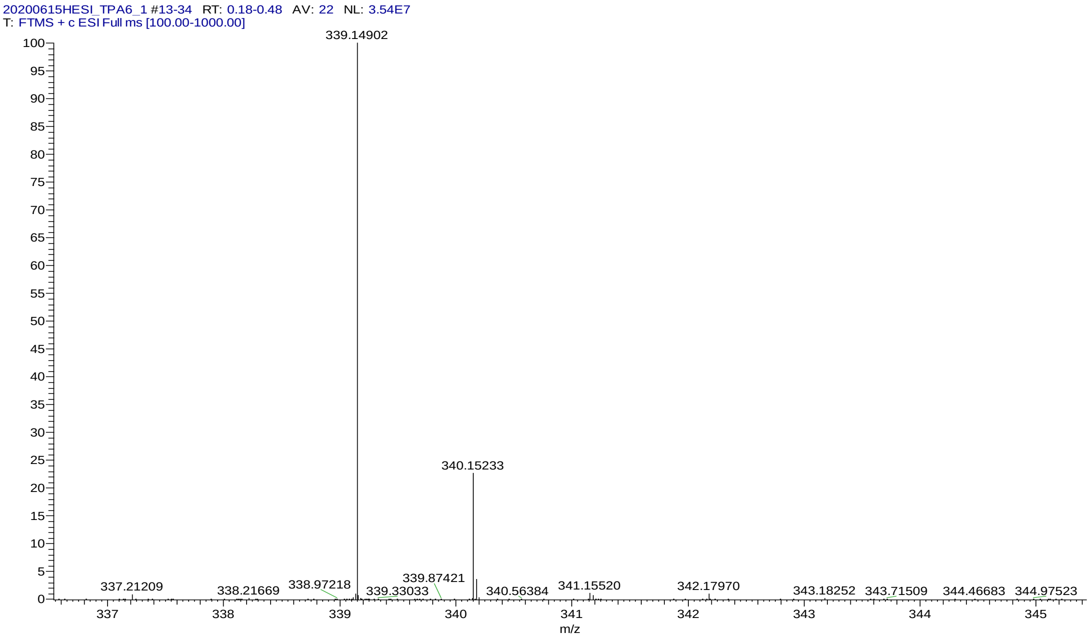

m/z	Intensity	Relative	Theo. Mass	Delta (mmu)	RDB equiv.	Composition
339.149	35421300	100	339.1492	-0.17	15.5	C23 H19 O N2
340.152	8024740	22.66	340.1525	-0.22	15.5	C22 [13]C H19 O N2

Supplementary Figure 31. ESI mass spectrum of **TPA6**.

References

1. Stephens, P. J., Devlin, F. J., Chabalowskim C. F. & Frisch, M. J. Ab initio calculation of vibrational absorption and circular dichroism spectra using density functional force fields. *J. Phys. Chem.* **98**,

11623-11627 (1994).

2. a) Grimme, S., Antony, J., Ehrlich, S. & Krieg, H. A consistent and accurate ab initio parametrization of density functional dispersion correction (DFT-D) for the 94 elements H-Pu. *J. Chem. Phys.* **132**, 154104 (2010); b) Grimme, S., Ehrlichm S. & Goerigkm L. Effect of the damping function in dispersion corrected density functional theory. *J Comput Chem* **32**, 1456-1465 (2011).
3. a) Hehre, W. J., Ditchfield, R. & Pople, J. A. Self-consistent molecular orbital methods. XII. Further extensions of Gaussian-type basis sets for use in molecular orbital studies of organic molecules. *J. Chem. Phys.* **56**, 2257-2261 (1972); b) Hariharan, P. C. & Pople, J. A. The influence of polarization functions on molecular orbital hydrogenation energies. *Theor. Chim. Acta* **28**, 213-222 (1973); c) Ditchfield, R., Hehre, W. J. & Pople, J. A. Self-consistent molecular-orbital methods. IX. An extended Gaussian-type basis for molecular-orbital studies of organic molecules. *J. Chem. Phys.* **54**, 724-728 (1971).
4. Vydrov, O. A. & Scuseria, G. E. Assessment of a long-range corrected hybrid functional. *J. Chem. Phys.* **125**, 234109 (2006).
5. Schäfer, A., Huber, C. & Ahlrichs, R. Fully optimized contracted Gaussian basis sets of triple zeta valence quality for atoms Li to Kr. *J. Chem. Phys.* **100**, 5829-5835 (1994)..
6. Tomasi, J., Mennucci, B. & Cammi, R. Quantum mechanical continuum solvation models. *Chem. Rev.* **105**, 2999-3094 (2005).
7. Lu, T. & Chen, F. Multiwfn: a multifunctional wavefunction analyzer. *J. Comput. Chem.* **33**, 580-592 (2012).
8. Humphrey, W., Dalke, A. & Schulten, K. VMD: visual molecular dynamics. *J. Mol. Graph.* **14**, 33-38 (1996).
9. Yanai, T., Tew, D. P. & Handy, N. C. A new hybrid exchange–correlation functional using the Coulomb-attenuating method (CAM-B3LYP). *Chem. Phys. Lett.* **393**, 51-57 (2004).
10. a) Heß, B. A. Marian, C. M. Wahlgren, U. & Gropen, O. A mean-field spin-orbit method applicable to correlated wavefunctions. *Chem. Phys. Lett.* **251**, 365-371 (1996); b) Neese F. Efficient and accurate approximations to the molecular spin-orbit coupling operator and their use in molecular g-tensor calculations. *J. Phys. Chem.* **122**, 034107 (2005).
11. Lin, Y. -S., Li, G. -D., Mao, S. -P. & Chai, J. -D. Long-range corrected hybrid density functionals with improved dispersion corrections. *J. Chem. Theory Comput.* **9**, 263-272 (2013).
12. a) Neese, F. The ORCA program system. *WIREs Comput. Mol. Sci.* **2**, 73-78 (2012); b) Neese, F. Software update: the ORCA program system, version 4.0. *WIREs Comput. Mol. Sci.* **8**, e1327 (2018).
13. Zhao, Y. & Truhlar, D. G. The M06 suite of density functionals for main group thermochemistry, thermochemical kinetics, noncovalent interactions, excited states, and transition elements: two new functionals and systematic testing of four M06-class functionals and 12 other functionals. *Theor.*

Chem. Acc. **120**, 215-241 (2008).

14. Goerigk, L. & Grimme, S. Efficient and Accurate Double-Hybrid-Meta-GGA Density Functionals-Evaluation with the Extended GMTKN30 Database for General Main Group Thermochemistry, Kinetics, and Noncovalent Interactions. *J. Chem. Theor. Comput.* **7**, 291-309 (2011).
15. Weigend, F. & Ahlrichs, R. Balanced basis sets of split valence, triple zeta valence and quadruple zeta valence quality for H to Rn: Design and assessment of accuracy. *Phys. Chem. Chem. Phys.* **7**, 3297-3305 (2005).
16. a) Neese, F. An improvement of the resolution of the identity approximation for the formation of the Coulomb matrix. *J. Comput. Chem.* **24**, 1740-1747 (2003). b) Neese, F., Wennmohs, F., Hansen, A. & Becker, U. Efficient, approximate and parallel Hartree–Fock and hybrid DFT calculations. A ‘chain-of-spheres’ algorithm for the Hartree–Fock exchange. *Chem. Phys.* **356**, 98-109 (2009).
17. Weigend, F. Accurate Coulomb-fitting basis sets for H to Rn. *Phys. Chem. Chem. Phys.* **8**, 1057-1065 (2006).
18. Jacquemin, D., Mennucci, B. & Adamo, C. Excited-state calculations with TD-DFT: from benchmarks to simulations in complex environments. *Phys. Chem. Chem. Phys.* **13**, 16987-16998 (2011).
19. Dunning, Jr T. H. Gaussian basis sets for use in correlated molecular calculations. I. The atoms boron through neon and hydrogen. *J. Chem. Phys.* **90**, 1007-1023 (1989).
20. Frisch, M. J. *et al.* Gaussian 16 Rev. A.03. (2016).
21. Sun, H., Zhong, C. & Bredas, J. -L. Reliable prediction with tuned range-separated functionals of the singlet–triplet gap in organic emitters for thermally activated delayed fluorescence. *J. Chem. Theory Comput.* **11**, 3851-3858 (2015).
